# Endothelial Progenitor Cells, Cardiovascular Risk Factors, Cytokine Levels and Atherosclerosis – Results from a Large Population-Based Study

**DOI:** 10.1371/journal.pone.0000975

**Published:** 2007-10-10

**Authors:** Qingzhong Xiao, Stefan Kiechl, Seema Patel, Friedrich Oberhollenzer, Siegfried Weger, Agnes Mayr, Bernhard Metzler, Markus Reindl, Yanhua Hu, Johann Willeit, Qingbo Xu

**Affiliations:** 1 Cardiovascular Division, King's College London, University of London, London, United Kingdom; 2 Department of Neurology, Medical University Innsbruck, Innsbruck, Austria; 3 Department of Internal Medicine, Hospital of Bruneck, Bruneck, Italy; 4 Department of Laboratory Medicine, Hospital of Bruneck, Bruneck, Italy; 5 Department of Internal Medicine, Medical University Innsbruck, Innsbruck, Austria; Monash University, Australia

## Abstract

**Background:**

EPC number and functionality are assumed to reflect the endogenous vascular repair capacity with the EPC pool declining in higher ages and being exhausted by unfavorable life-style and risk factors. This intriguing and clinically highly relevant concept, however, has so far been derived from small case-control studies and patient series.

**Methodology and Principle Findings:**

In the population-based Bruneck Study EPC number and EPC-colony forming units (EPC-CFU) were assessed as part of the fourth follow-up evaluation (2005) in 571 and 542 subjects, respectively. EPC number declined with age (p = 0.013), was significantly lower in women (p = 0.006) and higher in subjects on statin, hormone replacement or ACE inhibitor/angiotensin-receptor blockers, and correlated positively with moderate alcohol consumption. Unexpectedly, a positive relation between EPC number and several vascular risk factors emerged. In a step forward multivariate linear regression analysis EPC number was independently related with SDF1α, MMP-9, triglycerides, alcohol consumption, and Hba1c. EPC-CFU in turn was related to SDF1α and diastolic blood pressure. Moreover, EPC number showed a significant positive association with the Framingham risk score (P = 0.001). Finally, there was an inverse association between EPC number and common carotid artery intima-media thickness (p = 0.02) and the carotid artery atherosclerosis score (p = 0.059).

**Conclusions:**

Our population-based data confirm the decline of EPC number with advancing age and lend first epidemiological support to a role of SDF-1α and MMP9 in EPC differentiation, mobilization and homing, but are conflict with the view that EPC number is unfavorably affected by cardiovascular risk factors. EPC number increases with the cardiovascular risk estimated by the Framingham risk score (FRS), which in the absence of similar changes for EPC-CFU. Finally, we demonstrate a significant inverse association between EPC number and extent of carotid atherosclerosis even though this association was only of moderate strength and not entirely consistent in other vascular territories.

## Introduction

Asahara and colleagues first isolated circulating angioblasts from human peripheral blood, which had the potential to differentiate in vitro into endothelial cells and to contribute to neoangiogenesis after tissue ischemia in vivo, and defined this cell population as endothelial progenitor cells (EPC)[1], [2]. The mostly used methods to define EPC are the identification of mononuclear cell population expressing CD34, KDR/VEGFR2, and CD133/AC133 with adherent growth characteristics, whereas the function and the clonogenic capacity of EPC are evaluated using colony-forming units (EPC-CFU) assays[3], [4].

Functionally, it is believed that EPC play an important role in regeneration of ischemic and damaged tissues via angiogenesis and repairing denuded endothelium in the injured vessels[5]–[7]. It was demonstrated that reduced numbers of EPC predict future cardiovascular events and proposed that low EPC number and EPC-CFU reflect an impaired endogenous repair capacity[8], [9]. Of particular note, circulating EPC are believed to be depleted by standard cardiovascular risk factors and unfavorable life-style, and concerns have been expressed that this may restrict the therapeutic potential of progenitor cells[10]. Actually, several case-control studies and evaluations in patient series have demonstrated inverse associations between EPC number and age[11], diabetes[12], smoking[13], hypertension[14], family history for coronary artery disease[12], CRP leve[15], physical inactivity[16] and the Framingham risk score[4]. Evidence, however, is far from consistent with several studies failing to obtain such relations (especially after controlling for age) and some even reporting the opposite. For example, two recent studies including the largest available obtained a significant positive association between EPC number and smoking[8] or some risk factors in baseline level, such as arterial hypertension, hyperlipidemia, diabetes, family history of coronary artery diseases (CAD), and bod-mass index[9]. Furthermore, there is still a disturbing lack of in-depth insights into the mechanisms controlling EPC mobilization and turn over in humans. Further experimental and epidemiological studies are required to resolve all the controversies surrounding this intriguing issue. The current study is the first large scale evaluation in the general community and aims at further elaborating the association of EPC number and EPC-CFU with cardiovascular risk factors and life-style behaviors. An additional focus will be on the potential relation of EPC characteristics with atherosclerosis as well as levels of cytokines and growth factors previously implicated in EPC differentiation[17], mobilization[18], [19] and homing[20], [21].

## Methods

### Study Population

Population recruitment was performed as part of the Bruneck Study[22]. The survey area was located in the north of Italy (Bolzano Province). Special features of the study design and protocol have been described previously in detail[22]. The current study focused on the follow-up in 2005. EPC number and EPC-CFU were assessed in 571 and 542 of the 574 participants. Subjects with and without EPC number and EPC-CFU assessments did not differ in age, sex and expression of cardiovascular risk factors. The appropriate ethics committees (Autonome Provinz Bozen-Sanitatsbetrieb Bozen Ethikkomittee) approved the study protocol and all study subjects gave their written informed consent before entering the study.

### EPC Culture assay (EPC number)

PBMNC isolation from Venous blood and EPC identification were performed as previously reported[23]–[27]. Double positive stained cells for DiI-Ac-LDL and Lectin were considered as EPC on day 5 of culture. The total numbers of EPC per well were counted by two trained independent senior investigators blinded to the clinical details of the subjects. The EPC in a minimum of two wells were counted and the average was then recorded. Reproducibility was assessed over 50 samples in the present study by comparing the EPC numbers from the two individuals. Coefficient of variance was <10% in each case.

### EPC-CFU assay

The EPC-CFU assay performed as described previously[4], [12], [28]. PBMNC were resuspended in EPC culture medium (M199 with 20%FCS and antibiotics) and then plated on fibronectin coated 6 well plates at a concentration of 5 million cells per well. The endothelial colonies were counted manually on day 7. Strict guidelines were followed to ensure consistent counting of EPC colonies. Two senior investigators who were blinded to the subjects' clinical status counted colonies. Reproducibility was assessed over 50 samples in the present study comparing colony counts by the two individuals. Coefficient of variance was <10% in each case.

### Reproducibility assay

To assess reproducibility, the cEPC and EPC-CFU numbers were determined twice in two separate blood samples obtained at least one week apart from 10 subjects in our preliminary experiment. The samples were analyzed independently by two observers who were blinded to subjects' clinical profiles. The interobserver correlations were 0.965 and 0.975 for cEPC and EPC-CFU assay, whereas the intraclass correlations obtained from a single observer who analyzed two blood samples collected at least one week apart from 10 subjects, were 0.939 and 0.961, respectively.

### Enzyme-linked immunosorbent assay (ELISA)

Plasma granulocyte-colony stimulating factor (G-CSF), stromal cell-derived factor-1α (SDF1-α), vascular endothelial growth factor (VEGF) and matrix metalloproteinase-9 (MMP-9) levels were determined using commercially available kits (Quantikine, R&D Systems, UK). All ELISA tests were carried out at room temperature on freshly thawed plasma samples. The concentration of all cytokines was determined by comparison with a standard curve, following manufacturer's instruction. Other laboratory parameters were all examined by standard methods[29].

### Scanning Protocol and definition of Ultrasound End Points

The ultrasound protocol involves the scanning of the internal (bulbous and distal segments) and common carotid arteries (proximal and distal segments) of either side with a 10-MHz imaging probe[30]. The intima-media thickness (IMT) was quantified at the far wall of plaque-free sections of the common carotid arteries as the distance between the lumen-intima and media-adventitia interface (intra-observer coefficient of variation, 7.9 percent (n = 100))[30].

### Statistical Analysis

The data were analyzed using the SPSS 12.0 and BMDP software packages. Levels of variables according to tertile groups for EPC number and EPC-CFU were presented as mean values±SD or as medians with corresponding 25th and 75th percentiles (continuous variables), and as absolute numbers and percentages (dichotomous variables). Associations of EPC number and EPC-CFU tertile groups with vascular risk factors, life-style and demographic variables, IMT and severity of atherosclerosis were assessed using generalized linear models and logistic regression analysis. We performed tests for linear trend by treating the medians in each category of EPC number and EPC-CFU as a continuous variable. Levels of C-reactive protein, lipoprotein (a), D-dimer, triglycerides and urinary ACR were log_e_-transformed to satisfy the assumption of normality and constant variance of the residuals. The association between EPC number and EPC-CFU and IMT and the carotid atherosclerosis score was tested by means of linear regression analysis. The multivariate models included the variables age (years), sex (female, male), smoking (number of cigarettes smoked daily), alcohol consumption (g/day), diabetes (no, yes), body mass index (kg/m^2^), HDL and LDL cholesterol (mg/dL), log_e_-transformed triglycerides (mg/dL), log_e_-transformed hs-CRP level (mg/L), log_e_-transformed Lp(a) level (mg/dL), log_e_-transformed urinary ACR (ratio), ferritin concentration (µg/L), systolic blood pressure (mmHg) and facultatively various types of medication. A two-sided p value <0.05 was considered significant.

## Results

### Measurement and distribution of EPC and EPC colony

Detailed Characterization of EPC in colony formation and quantification assay was carried out in present study (**Text S1 and figure S1**). The EPC number in circulation was investigated in all the 571 subjects, but only 542 subjects were subject to EPC colony analysis due to the limitation of blood sample and PBMNC number in 29 subjects. In this study, we have determined the EPC number and EPC-CFU number by adjusting to per 10^6^ PBMC and per 1 ml of blood, to avoid any discrepancy caused by total circulating PBMNC variation happened in some situation. The later data (per 1 ml of blood) are summarized in the online supplementary data, which are very similar for most aspects to former data (per 10^6^ PBMC) (**Data not shown)**. F**igure S2A** show the non-normal distribution of EPC number and EPC-CFU number (per 10^6^ PBMC), which indicate that the EPC and EPC-CFU numbers from a majority of subjects (higher than 70%) fall into (0-1000 EPC) and (0-250 EPC-CFU) score groups, respectively. F**igure S2B** illustrates a moderate but significant decline of EPC number (P = 0.013) but not EPC-CFU number (per 10^6^ PBMC) with age.

### Associations of EPC and EPC-CFU number with cardiovascular risk factors and other variables

**Table 1** described the associations of demographic and life-style characteristics, vascular risk factors, laboratory parameters and medications with EPC number in circulation. EPC number was significantly lower in female subjects (p = 0.006). It can be clearly seen that there was no inverse correlation between levels of various risk factors and EPC number. On the contrary, some risk factors showed positive correlations, for instance hypertension (p = 0.009), Hba1c level (p = 0.016) and triglycerides (p = 0.001). The positive correlations between EPC number and hypertension, Hba1c and triglycerides remain significant even when restricting the analyses to subjects without any medication, which indicates that these unexpected trends are not confounded by drug therapy. In addition, there is a positive correlation with alcohol consumption (p = 0.014) and with some types of medications (statins, hormone replacement and ACE inhibitors/angiotensin-receptor blockers). However, no clear correlation between EPC number and cardiovascular disease history was found. **Table 2** describes the associations of demographic and life-style characteristics, vascular risk factors, laboratory parameters and medications with EPC-CFU number. There was no clear correlation observed between EPC-CFU number and levels of risk factors or other variables except for a weak correlation with diastolic blood pressure (p = 0.017).

**Table 1:** Association of demographic and life-style characteristics, vascular risk factors, laboratory parameters and medication with EPC number (n = 571)

Characteristic *	tertile group for epc number			P Value for Trend†
	Low EPC level (n = 190)	Medium EPC level (n = 190)	High EPC level (n = 191)	
**EPC number (per 10^6^ PBMNC)**				
Median	127	560	1386	
Range	17–295	296–859	860–4768	
**Demographic variables**				
Age (years)	71.2±10.2	67.5±9.2	68.9±8.9	0.072
Sex (%)				0.006
Men	40.0	45.3	53.9	
Women	60.0	54.7	46.1	
**Life-style and vascular risk variables**				
Smoking (%)	14.7	11.1	15.2	0.995
Smoking (cigarettes per day)	1.5±4.3	1.3±4.3	1.9±5.4	0.646
Alcohol consumption (g/day)	13.5±20.6	18.2±26.8	22.6±27.9	0.014
Physical activity (Sports score)	2.3±0.7	2.4±0.7	2.4±0.7	0.892
Diabetes mellitus (%)	10.5	6.8	13.6	0.109
Fasting glucose (mg/dL)	102.1±25.0	100.2±14.1	106.2±25.7	0.050
Hba1_c_ (%)	5.7±0.7	5.6±0.4	5.9±0.8	0.016
Waist (cm)	90.8±12.2	90.9±12.6	92.2±11.8	0.727
Body-mass index (kg/m^2^)	25.9±4.2	25.9±4.2	26.1±4.1	0.715
HDL cholestrerol (mg/dL)	62.3±13.5	67.2±14.3	62.8±13.8	0.639
LDL cholestrerol (mg/dL)	134.0±31.4	140.9±33.3	132.5±37.4	0.705
Triglycerides (mg/dL)	112 (91–151)	114 (91–152)	124 (92–181)	0.001
Lipoprotein(a) (mg/dL)	15.1 (5.4–37.1)	14.4 (6.4–46.9)	12.9 (4.8–40.9)	0.810
High-sensitivity CRP (mg/L)	2.1 (1.0–4.5)	1.8 (1.0–4.0)	2.1 (1.3–4.7)	0.135
Urinary ACR (mg/g)	6.9 (3.9–16.3)	6.9 (4.0–13.1)	6.5 (3.9–15.6)	0.419
Hypertension (%)	69.5	58.4	76.4	0.009
Systolic blood pressure (mmHg)	141.1±17.9	140.0±21.6	143.2±20.6	0.123
Diastolic blood pressure (mmHg)	83.7±8.0	83.1±9.3	83.2±8.9	0.633
Creatinine (mg/dL)	1.0±0.2	1.0±0.2	1.0±0.1	0.442
Fibrinogen (mg/dL)	304.0±64.5	293.1±55.2	297.7±57.0	0.917
Antithrombin III (%)	96.1±11.7	98.2±13.6	96.3±13.2	0.621
D-dimer (mg/L)	0.7 (0.4–1.0)	0.5 (0.3–0.8)	0.5 (0.3–0.8)	0.231
Ferritin (µg/L)	108.9±101.8	113.2±120.1	138.0±146.3	0.093
Homocystein (µmol/L)	12.5±6.1	12.0±6.4	12.3±6.0	0.931
**Medication (%)**				
Statins	13.7	14.2	22.5	0.005
Aspirin	21.6	19.5	25.7	0.063
ACE inhibitors/ARBs	24.7	23.2	31.4	0.031
Beta-blockers	13.7	11.1	16.8	0.119
Calcium-channel blockers	14.7	7.9	17.3	0.104
Diuretics	24.2	16.8	25.7	0.084
Digitalis	5.3	5.3	6.3	0.152
Corticosteroids	0.5	0.5	0.5	0.554
Cumarine	2.1	6.3	4.2	0.418
Bisphosphonates	6.3	6.3	5.2	0.498
NSAR	12.6	6.8	11.5	0.793
Sex hormones‡	5.3	6.7	13.6	0.041
**Previous cardiovascular disease (%)**				
Ischemic stroke, myocardial infarction, symptomatic peripheral artery disease and/or any revascularisation procedure	10.5	10.5	11.5	0.637

ARBs, angiotensin-receptor blockers.

* Values presented are unadjusted means±SD, medians (IQR) or percentages.

† P values for the variables age and sex are from unadjusted analyses. All other P values are from analyses adjusted for age and sex.

‡ Analysis was confined to women only.

Factors to convert conventional units into SI units are as follows: glucose (mmol/L, 0.05551); cholestrerol (mmol/L, 0.0259); Triglycerides (mmol/L, 0.01129); Lipoprotein (a) (µmol/L, 0.0357); Creatinine (µmol/L, 72.26); Fibrinogen (µmol/L, 0.0294); Ferritin (pmol/L, 2.247).

**Table 2:** Association of demographic and life-style characteristics, vascular risk factors, laboratory parameters and medication with EPC-CFUs number (n = 542)

Characteristic *	tertile group for epc number			P Value for Trend†
	Low EPC colony number (n = 180)	Medium colony number (n = 181)	High colony number (n = 181)	
**EPC-CFUs (per 10^6^ PBMNC)**				
Median	0	91	407	
Range	0–23	24–202	203–1032	
**Demographic variables**				
Age (years)	68.9±9.6	68.3±9.6	68.9±9.3	0.850
Sex (%)				0.278
Men	43.9	46.4	49.7	
Women	56.1	53.6	50.3	
**Life-style and vascular risk variables**				
Smoking (%)	12.8	14.9	14.4	0.811
Smoking (cigarettes per day)	1.5±4.7	1.6±4.7	1.6±4.8	0.992
Alcohol consumption (g/day)	17.7±24.8	15.8±23.7	20.5±27.7	0.304
Physical activity (Sports score)	2.3±0.7	2.4±0.7	2.4±0.7	0.410
Diabetes mellitus (%)	11.1	8.8	11.0	0.812
Fasting glucose (mg/dL)	104.3±23.8	102.4±25.4	101.6±17.5	0.248
Hba1_c_ (%)	5.7±0.7	5.7±0.7	5.8±0.7	0.370
Waist (cm)	92.3±11.5	91.0±12.0	91.1±13.1	0.237
Body-mass index (kg/m^2^)	26.3±3.9	25.8±4.2	25.9±4.4	0.534
HDL cholestrerol (mg/dL)	64.2±13.7	64.6±13.6	63.6±14.7	0.741
LDL cholestrerol (mg/dL)	138.3±34.9	136.6±31.3	134.1±36.1	0.350
Triglycerides (mg/dL)	120 (91–163)	110 (93–155)	122 (92–158)	0.488
Lipoprotein(a) (mg/dL)	14.4 (4.5–36.7)	14.9 (6.3–45.7)	14.8 (5.8–41.3)	0.638
High-sensitivity CRP (mg/L)	1.9 (1.0–3.8)	1.8 (1.0–4.8)	2.1 (1.3–4.3)	0.242
Urinary ACR (mg/g)	6.6 (3.7–13.3)	7.1 (4.3–16.8)	6.5 (3.8–14.1)	0.821
Hypertension (%)	70.6	66.3	66.9	0.570
Systolic blood pressure (mmHg)	142.2±19.2	141.3±21.7	140.5±19.5	0.482
Diastolic blood pressure (mmHg)	84.5±8.2	83.4±9.6	82.1±8.4	0.017
Creatinine (mg/dL)	1.0±0.2	1.0±0.2	1.0±0.2	0.789
Fibrinogen (mg/dL)	294.5±63.5	299.3±57.3	298.1±56.5	0.554
Antithrombin III (%)	95.8±11.9	96.9±13.0	98.0±13.3	0.049
D-dimer (mg/L)	0.6 (0.3–0.9)	0.5 (0.3–0.9)	0.5 (0.3–0.8)	0.128
Ferritin (µg/L)	121.4±139.5	110.9±96.6	127.7±136.6	0.605
Homocystein (µmol/L)	12.7±6.0	11.9±6.3	12.3±6.4	0.746
**Medication (%)**				
Statins	12.2	22.1	16.6	0.681
Aspirin	18.9	23.2	23.8	0.302
ACE inhibitors/ARBs	24.4	22.1	32.0	0.038
Beta-blockers	11.7	11.0	18.2	0.024
Calcium-channel blockers	11.7	12.7	13.3	0.657
Diuretics	20.0	20.4	24.3	0.189
Digitalis	2.2	5.5	7.7	0.018
Corticosteroids	0.0	0.6	1.1	0.180
Cumarine	3.3	4.4	5.0	0.495
Bisphosphonates	6.1	5.5	6.1	0.702
NSAR	9.4	11.0	10.5	0.761
Sex hormones‡	8.9	7.2	8.8	0.908
**Previous cardiovascular disease (%)**				
Ischemic stroke, myocardial infarction, symptomatic peripheral artery disease and/or any revascularisation procedure	7.2	13.8	12.2	0.330

* Values presented are unadjusted means±SD, medians (IQR) or percentages.

† P values for the variables age and sex are from unadjusted analyses. All other P values are from analyses adjusted for age and sex.

‡ Analysis was confined to women only.

Factors to convert conventional units into SI units are as indicated in Table 1.

### The EPC numbers, but not EPC-CFU, are positively correlated with the FRS

**Figure 1:**
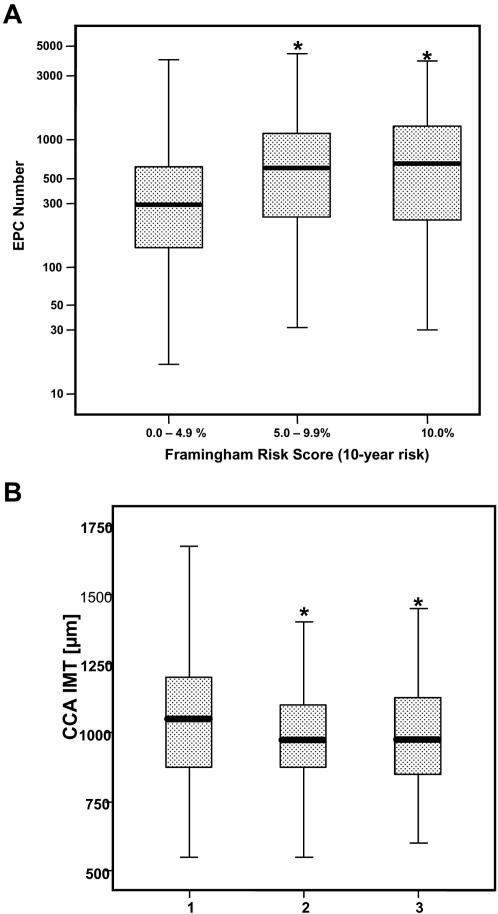
Panel A illustrates the association between EPC number (per 10^6^ PBMNC) and the FRS (p = 0.001). Panel B illustrates the association between IMT and EPC number (per 10^6^ PBMNC) (p = 0.02).

**Figure 1A** shows the association between EPC number and the FRS (10-years risk). Surprisingly, a positive association between EPC number and the FRS emerged (P = 0.001). There was no differential association in men and women and significance was maintained when adjusting for the use of statins, hormone replacement therapy, ACE inhibitors and angiotensin receptor blockers all of which may affect levels of circulating EPC. However, we did not find any association between EPC-CFU number and the FRS (**data not shown**).

### Inverse correlation of EPC but not EPC-CFU with common carotid artery atherosclerosis

**Table S1 and** **Figure 1B** illustrated the associations of EPC and EPC-CFU number with common carotid artery intima-media thickness (CCA IMT) and the carotid artery atherosclerosis score. EPC number but not EPC-CFU were inversely associated with CCA IMT (p = 0.02) and the atherosclerosis score (P = 0.059). In the femoral arteries, however, inverse trends of association between EPC number and both IMT and the atherosclerosis score clearly fell short of statistical significance.

### The strongest determinant of EPC and EPC-CFU number and function is the SDF-1α level

**Table 3** **and** **4** show the associations of progenitor related cytokines with EPC number and EPC-CFU number. It is apparent that plasma SDF-1α levels were significantly and inversely related to EPC number (p = 0.02) and EPC-CFU number (p = 0.013). However, there are no significant correlations between EPC or EPC-CFU number and VEGF, MMP-9, and G-CSF levels in the univariate analysis. In a step forward multivariate linear regression analysis allowing for all variables in Tables 1 and 4, log_e_-transformed EPC number was independently related with log_e_-transformed SDF-1α (inverse relation, P<0.001), log_e_-transformed MMP-9 (P = 0.005), log_e_-transformed triglycerides (P = 0.009), alcohol consumption (P = 0.002) and Hba1c (P = 0.052). Log_e_-transformed EPC-CFU number in turn was related to log_e_-transformed SDF-1α and diastolic blood pressure (both inverse, P = 0.001 and P = 0.007).

**Table 3:** Association of cytokines levels with EPC number (n = 571)

Characteristic *	tertile group for epc number			P Value for Trend†
	Low EPC level (n = 190)	Medium EPC level (n = 190)	High EPC level (n = 191)	
**EPC number (per 10^6^ PBMNC)**				
Median	127	560	1386	
Range	17–295	296–859	860–4768	
**cytokines**				
GCSF (pg/ml)	10.3 (4.2–18.3)	8.2 (3.6–14.5)	9.9 (5.0–17.8)	0.202
MMP9 (ng/ml)	57.8 (32.1–111.0)	66.3 (38.0–105.0)	71.4 (37.2–113.0)	0.295
SDF-1 (pg/ml)	2619 (2311–2956)	2469 (2218–2834)	2503 (2228–2767)	0.020
VEGF (pg/ml)	70.9 (30.0–138.8)	77.9 (34.5–162.9)	73.2 (27.5–158.9)	0.574

* Values presented are unadjusted means±SD or medians (IQR). Levels for GCSF, MMP9 and VEGF were available in 264, 556 and 374 subjects.

† P values are from analyses adjusted for age and sex.

**Table 4:** Association of cytokines with EPC colonY number (n = 542)

Characteristic *	tertile group for epc-cfu number			P Value for Trend†
	Low EPC colony number (n = 180)	Medium EPC colony number (n = 181)	High EPC colony number (n = 181)	
**EPC-CFU (per 10^6^ PBMNC)**				
Median	0	91	407	
Range	0–23	24–202	203–1032	
**cytokines**				
GCSF (pg/ml)	10.8 (5.1–18.7)	8.4 (5.1–13.5)	8.9 (4.2–17.4)	0.655
MMP9 (ng/ml)	63.8 (32.0–113.2)	61.6 (33.5–96.0)	68.1 (37.0–118.7)	0.605
SDF-1 (pg/ml)	2554 (2269–2922)	2553 (2292–2857)	2486 (2196–2769)	0.013
VEGF (pg/ml)	72.6 (35.6–145.1)	96.8 (29.3–163.8)	58.2 (24.2–135.6)	0.325

* Values presented are unadjusted means±SD or medians (IQR). Levels for GCSF, MMP9 and VEGF were available in 249, 528 and 356 subjects.

† P values are from analyses adjusted for age and sex.

## Discussion

Our study lends further support to the previous observations that EPC number declines with advancing age and is influenced by standard cardiovascular drugs, refutes the concept that the circulating EPC pool is exhausted and repair capacity impaired by vascular risk factors and an unfavorable life-style and reports important novel findings like the significant association between EPC number and cytokine levels as well as carotid artery IMT.

Most important consideration in EPC experiment is the methods to choice. Various surface markers are expressed on EPCs and are used for EPC characterization[3], for example CD34, vascular endothelial growth factor receptor 2 (VEGFR2) or kinase domain receptor (KDR). Further studies indicated that there is an immature subset of EPCs which expresses the surface marker AC133 or CD133 and share more characteristics of stem/progenitor cells[3], [31], [32]. The ability of peripheral blood-derived EPCs to form “late-outgrowth colony-forming units–ECs” underlines their stem cell–like properties and gives information about the clonogenic potential of these cells. This cell heterogeneity may apparently reflect different developmental stages of EPCs during the maturational process from the bone marrow residual cell to the mature vascular wall cell. So far, the most used methods to define EPCs are identifying mononuclear cell population expressing CD34, KDR/VEGFR2, and CD133/AC133 with adherent growth characteristics, and measuring the circulating numbers of EPCs by flow cytometry using either antibodies against CD34 and KDR or CD133. Whereas the function and the clonogenic capacity of EPC should be evaluated using colony-forming unit (EPC-CFUs) assays[3], [4]. The methods to measure the circulating numbers of EPCs by flow cytometry using either antibodies against CD34 and KDR or CD133 were used in many previous studies. However, very strict and rigorous technology in flow cytometry analysis is needed and a completed negative/positive control should be included in this analysis, because it is very difficult to reproduce the data of EPC numbers due to the limitation of non-specific background of this measurement and just a very small fraction of EPCs existed in the circulation. Alternatively, phenotypes of human EPCs may be confirmed by uptake of 1, 1′-dioctadecyl-3, 3′ 3′-tetramethylindo-carbocyanine-labeled acetyl low-density lipoprotein (DiI-Ac-LDL) and binding of ulex-lectin[33]. Many important studies have shown that EPCs could be characterized and identified by dual-staining for DiI-Ac-LDL, lectin, and expression of endothelial markers KDR, VE-cadherin, and vWF[23], [25]–[27]. According to our preliminary data and previous publications[23]–[27], double positive cells for DiI-Ac-LDL and Lectin were counted as cEPCs and used in the present study to analyze the EPC numbers in circulation.

Obviously, our findings are different compared to the other published clinical studies in cardiovascular disease. As mentioned above, such discrepancy may be due to different methods used to characterise and quantify putative EPCs, preventing straightforward comparisons between them and probably explaining the difference in the results. The methods to measure the circulating numbers of EPCs by flow cytometry using either antibodies against CD34 and KDR or CD133 were used in many previous studies. However, double positive cells for DiI-Ac-LDL and Lectin in EPC culture were counted as EPCs and used in the present study to analyze the EPC numbers in circulation. The advantage for our method is that the number of cultured EPCs not only reflect the levels of circulating EPCs in humans, but also the proliferative potential of EPC which is responsible for EPC function in angiogenesis and vasculogenesis.

Another interesting observation in the current study is the number of cultured EPCs, but not EPC-CFUs, was associated with some cardiovascular risk factors, FRS and common carotid artery atherosclerosis. Since the number of cultured EPCs include total EPCs with lower or higher proliferative potential, whereas EPC-CFU assay assesses a proportion of EPCs with higher proliferative ability and clonogenic capacity. One possible explanation for such difference between the number of cultured EPCs and EPC-CFU assay is that they represent different functional subpopulation of circulating EPCs. As controversy still exists over the exact definition of circulating EPCs, comparison between studies using different technique to assess EPC must be considered.

Past studies were highly consistent in reporting inverse associations between EPC number and age[11], and postulated that this trend renders elderly people prone to endothelial dysfunction and cardiovascular diseases[11]. In the Bruneck cohort representative for the general community a modest but significant decline in the pool of circulating EPC emerged across the age range from 55 to 94 years (WEB Figure 2B). Moreover, we demonstrated for the first time that the EPC number is significantly lower in (predominantly postmenopausal) females than in males of equal age and higher among subjects reporting regular (predominantly low-to-moderate) alcohol consumption. In addition, subjects with statin, hormone replacement and ACE inhibitor/angiotensin-receptor blocker therapy exhibited higher EPC levels. This observation fits very well to previous reports showing that these standard cardiovascular drugs are capable of enhancing EPC numbers[34]–[36].

Several case-control studies and evaluations in patient series indicated that the EPC number is inversely associated with cardiovascular risk factors[4], [12], [13], [28], [37]–[40] giving raise to the view that EPC are exhausted by the risk burden and unfavourable life-style. However, the evidence available is far from consistent and some studies even reported significant positive associations between EPC and risk behaviors like smoking[8], [9]. In our study we revealed positive associations of EPC number with a number of cardiovascular risk factors (table 1) and with the Framingham risk score (FRS) (Figure 1A). The finding persisted after adjusting for statin, hormone replacement and ACE inhibitor/angiotensin-receptor blocker therapy. Speculatively, the positive association between EPC number and the FRS in our study may reflect a protective compensatory response to the individual vascular risk burden. Discrepancy between the results of our current study and previous various clinical studies may be explained by differences in study design, composition of the study populations and variations in risk factor levels. For instance, all the blood samples had been taken at the exact same time (7:00 to 8:00 in the early morning each day) and performed EPC analysis 2 hours later in our current study. Most recently, our preliminary experimental data implicated that the blood sample collection time for EPC analysis is particularly important. We observed that EPC number exhibited diurnal variation with a 40–50% increase between 3pm and 10pm and a 30–40% increase at 10pm compared with 8am. There are also implications for studies involving serial sampling, such as following myocardial infarction, where it is difficult to collect blood samples at the same time, i.e. at a specific time point. Finally it should be mentioned in this context that the current survey is the largest available and the only one representative of the general community. On the other hand the findings obtained to not necessarily extend to extreme expressions of risk factors which rarely occur in the general population.

Vascular repair is crucial to atherogenesis especially in its advanced stages in which plaque fissuring and endothelial denudation are common phenomena. Circulating EPCs have been suggested to crucially contribute to vascular repair. Rauscher and colleagues have explored the potential effects of EPC on vessel pathology in ApoE deficient mice[41]. They found that old ApoE deficient mice had suffered from exhaustive consumption of their EPC and this had promoted development of atherosclerosis by deficient vascular repair mechanism. Our study is the first to assess an inverse association between EPC number and common carotid artery IMT (p = 0.02) as well as the carotid artery atherosclerosis score (p = 0.059) both of which are surrogates for the severity of systemic vessel pathology. However, the associations obtained were only of moderate strength and did not extent to femoral artery atherosclerosis. Previous studies had obtained solid associations between severity of coronary artery disease and EPC number in symptomatic patients. Taken altogether these data indicate that EPC may protect against atherogenesis but also that their relevance increase with advancing vessel pathology and in symptomatic cardiovascular disease.

There is solid evidence from experimental studies that progenitor-related cytokines, such as VEGF[17], G-CSF[18], [19], SDF-1α[20], [21], MMP-9[42], [43] play a role in the EPC differentiation, mobilization into circulation and homing. In the present study we found a highly significant inverse relation between plasma SDF-1α levels and both EPC number and EPC-CFU and a significant positive relation between MMP-9 level and EPC number (multivariate models). It was postulated that SDF-1α has the ability to mediate homing of circulating EPC to tissues or organs to be repaired or maintained, which may explain the lower EPC pool in subjects with high SDF-1α. MMP-9 in turn was implicated in the release of EPC from bone marrow into blood[43]. There is a clear need for further investigating the mechanisms by which SDF-1 and MMP-9 are engaged in EPC mobilization and homing.

Counter expectation, in our study EPC number did not significantly differ between subjects with and without a history of previous cardiovascular disease. On interpreting these finding, however, it has to considered that most events had occurred already years before conduction of these study and that patients had been subject to extensive life-style modifications and drug therapy. Furthermore, many patients had again entered a clinically inactive stage of vessel disease. In contrast, the recent intriguing studies revealing lower levels of EPC in subjects with cardiovascular disease had a focus on patients with symptomatic coronary artery disease and acute coronary syndromes. In these patients differences in EPC number and functionality may be more pronounced because of an amplified consumption or inadequate mobilization of circulating EPC.

In summary, our data implicated that the changes of the number of EPCs are loosely associated with certain risk factors for the cardiovascular disease and may not directly associated with the disease development. In additional, one of our major finding in the current study is unfavourable to the traditional view that the EPC number is negatively affected by cardiovascular risk factors, indicating that the role of EPC is more complex than assumed previously. Our knowledge about the role of EPCs is far from complete and will require further revisions. Compared to previously data from case-control studies, our data also implicated that the capacity to mobilze EPCs in acute disease is more important than EPC basline levels.

## Supporting Information

Table S1 (0.06 MB DOC)

Text S1 (0.07 MB DOC)

Figure S1 Characterisation of EPC-CFU and EPC
(2.88 MB TIF)

Figure S2 Distribution of EPC and EPC-CFU, and the decline of EPC and EPC-CFU numbers with age.
(1.17 MB TIF)

## References

[1] Asahara T, Murohara T, Sullivan A, Silver M, van der Zee R (1997). Isolation of putative progenitor endothelial cells for angiogenesis.. Science.

[2] Asahara T, Masuda H, Takahashi T, Kalka C, Pastore C (1999). Bone marrow origin of endothelial progenitor cells responsible for postnatal vasculogenesis in physiological and pathological neovascularization.. Circ Res.

[3] Rafii S, Lyden D (2003). Therapeutic stem and progenitor cell transplantation for organ vascularization and regeneration.. Nat Med.

[4] Hill JM, Zalos G, Halcox JP, Schenke WH, Waclawiw MA (2003). Circulating endothelial progenitor cells, vascular function, and cardiovascular risk.. N Engl J Med.

[5] Kocher AA, Schuster MD, Szabolcs MJ, Takuma S, Burkhoff D (2001). Neovascularization of ischemic myocardium by human bone-marrow-derived angioblasts prevents cardiomyocyte apoptosis, reduces remodeling and improves cardiac function.. Nat Med.

[6] Kalka C, Masuda H, Takahashi T, Kalka-Moll WM, Silver M (2000). Transplantation of ex vivo expanded endothelial progenitor cells for therapeutic neovascularization.. Proc Natl Acad Sci U S A.

[7] Kawamoto A, Gwon HC, Iwaguro H, Yamaguchi JI, Uchida S (2001). Therapeutic potential of ex vivo expanded endothelial progenitor cells for myocardial ischemia.. Circulation.

[8] Kunz GA, Liang G, Cuculoski F, Gregg D, Vata KC (2006). Circulating endothelial progenitor cells predict coronary artery disease severity.. Am Heart J.

[9] Werner N, Kosiol S, Schiegl T, Ahlers P, Walenta K (2005). Circulating endothelial progenitor cells and cardiovascular outcomes.. N Engl J Med.

[10] Werner N, Nickenig G (2006). Influence of cardiovascular risk factors on endothelial progenitor cells: limitations for therapy?. Arterioscler Thromb Vasc Biol.

[11] Scheubel RJ, Zorn H, Silber RE, Kuss O, Morawietz H (2003). Age-dependent depression in circulating endothelial progenitor cells in patients undergoing coronary artery bypass grafting.. J Am Coll Cardiol.

[12] George J, Herz I, Goldstein E, Abashidze S, Deutch V (2003). Number and adhesive properties of circulating endothelial progenitor cells in patients with in-stent restenosis.. Arterioscler Thromb Vasc Biol.

[13] Kondo T, Hayashi M, Takeshita K, Numaguchi Y, Kobayashi K (2004). Smoking cessation rapidly increases circulating progenitor cells in peripheral blood in chronic smokers.. Arterioscler Thromb Vasc Biol.

[14] Imanishi T, Moriwaki C, Hano T, Nishio I (2005). Endothelial progenitor cell senescence is accelerated in both experimental hypertensive rats and patients with essential hypertension.. J Hypertens.

[15] Verma S, Kuliszewski MA, Li SH, Szmitko PE, Zucco L (2004). C-reactive protein attenuates endothelial progenitor cell survival, differentiation, and function: further evidence of a mechanistic link between C-reactive protein and cardiovascular disease.. Circulation.

[16] Laufs U, Werner N, Link A, Endres M, Wassmann S (2004). Physical training increases endothelial progenitor cells, inhibits neointima formation, and enhances angiogenesis.. Circulation.

[17] Young PP, Hofling AA, Sands MS (2002). VEGF increases engraftment of bone marrow-derived endothelial progenitor cells (EPCs) into vasculature of newborn murine recipients.. Proc Natl Acad Sci U S A.

[18] Powell TM, Paul JD, Hill JM, Thompson M, Benjamin M (2005). Granulocyte colony-stimulating factor mobilizes functional endothelial progenitor cells in patients with coronary artery disease.. Arterioscler Thromb Vasc Biol.

[19] Kong D, Melo LG, Gnecchi M, Zhang L, Mostoslavsky G (2004). Cytokine-induced mobilization of circulating endothelial progenitor cells enhances repair of injured arteries.. Circulation.

[20] De Falco E, Porcelli D, Torella AR, Straino S, Iachininoto MG (2004). SDF-1 involvement in endothelial phenotype and ischemia-induced recruitment of bone marrow progenitor cells.. Blood.

[21] Ceradini DJ, Kulkarni AR, Callaghan MJ, Tepper OM, Bastidas N (2004). Progenitor cell trafficking is regulated by hypoxic gradients through HIF-1 induction of SDF-1.. Nat Med.

[22] Xu Q, Willeit J, Marosi M, Kleindienst R, Oberhollenzer F (1993). Association of serum antibodies to heat-shock protein 65 with carotid atherosclerosis.. Lancet.

[23] Urbich C, Heeschen C, Aicher A, Dernbach E, Zeiher AM (2003). Relevance of monocytic features for neovascularization capacity of circulating endothelial progenitor cells.. Circulation.

[24] Vasa M, Fichtlscherer S, Adler K, Aicher A, Martin H (2001). Increase in Circulating Endothelial Progenitor Cells by Statin Therapy in Patients With Stable Coronary Artery Disease.. Circulation.

[25] Aicher A, Heeschen C, Mildner-Rihm C, Urbich C, Ihling C (2003). Essential role of endothelial nitric oxide synthase for mobilization of stem and progenitor cells.. Nat Med.

[26] Seeger FH, Haendeler J, Walter DH, Rochwalsky U, Reinhold J (2005). p38 mitogen-activated protein kinase downregulates endothelial progenitor cells.. Circulation.

[27] Chavakis E, Aicher A, Heeschen C, Sasaki K, Kaiser R (2005). Role of beta2-integrins for homing and neovascularization capacity of endothelial progenitor cells.. J Exp Med.

[28] Grisar J, Aletaha D, Steiner CW, Kapral T, Steiner S (2005). Depletion of endothelial progenitor cells in the peripheral blood of patients with rheumatoid arthritis.. Circulation.

[29] Xu Q, Schett G, Perschinka H, Mayr M, Egger G (2000). Serum soluble heat shock protein 60 is elevated in subjects with atherosclerosis in a general population.. Circulation.

[30] Kiechl S, Muigg A, Santer P, Mitterer M, Egger G (1999). Poor response to activated protein C as a prominent risk predictor of advanced atherosclerosis and arterial disease.. Circulation.

[31] Gill M, Dias S, Hattori K, Rivera ML, Hicklin D (2001). Vascular trauma induces rapid but transient mobilization of VEGFR2(+)AC133(+) endothelial precursor cells.. Circ Res.

[32] Handgretinger R, Gordon PR, Leimig T, Chen X, Buhring HJ (2003). Biology and plasticity of CD133+ hematopoietic stem cells.. Ann N Y Acad Sci.

[33] Rehman J, Li J, Orschell CM, March KL (2003). Peripheral blood “endothelial progenitor cells” are derived from monocyte/macrophages and secrete angiogenic growth factors.. Circulation.

[34] Dimmeler S, Aicher A, Vasa M, Mildner-Rihm C, Adler K (2001). HMG-CoA reductase inhibitors (statins) increase endothelial progenitor cells via the PI 3-kinase/Akt pathway.. J Clin Invest.

[35] Spyridopoulos I, Haendeler J, Urbich C, Brummendorf TH, Oh H (2004). Statins enhance migratory capacity by upregulation of the telomere repeat-binding factor TRF2 in endothelial progenitor cells.. Circulation.

[36] Levy BI (2005). Beneficial effects of circulating progenitor endothelial cells activated by angiotensin receptor antagonists.. Hypertension.

[37] Vasa M, Fichtlscherer S, Aicher A, Adler K, Urbich C (2001). Number and migratory activity of circulating endothelial progenitor cells inversely correlate with risk factors for coronary artery disease.. Circ Res.

[38] George J, Goldstein E, Abashidze S, Deutsch V, Shmilovich H (2004). Circulating endothelial progenitor cells in patients with unstable angina: association with systemic inflammation.. Eur Heart J.

[39] Ghani U, Shuaib A, Salam A, Nasir A, Shuaib U (2005). Endothelial progenitor cells during cerebrovascular disease.. Stroke.

[40] Choi JH, Kim KL, Huh W, Kim B, Byun J (2004). Decreased number and impaired angiogenic function of endothelial progenitor cells in patients with chronic renal failure.. Arterioscler Thromb Vasc Biol.

[41] Rauscher FM, Goldschmidt-Clermont PJ, Davis BH, Wang T, Gregg D (2003). Aging, progenitor cell exhaustion, and atherosclerosis.. Circulation.

[42] Yoon CH, Hur J, Park KW, Kim JH, Lee CS (2005). Synergistic neovascularization by mixed transplantation of early endothelial progenitor cells and late outgrowth endothelial cells: the role of angiogenic cytokines and matrix metalloproteinases.. Circulation.

[43] Iwakura A, Shastry S, Luedemann C, Hamada H, Kawamoto A (2006). Estradiol enhances recovery after myocardial infarction by augmenting incorporation of bone marrow-derived endothelial progenitor cells into sites of ischemia-induced neovascularization via endothelial nitric oxide synthase-mediated activation of matrix metalloproteinase-9.. Circulation.

