# Supplementary material for: Endothelial Progenitor Cells, Cardiovascular Risk Factors, Cytokine Levels and Atherosclerosis – Results from a Large Population-Based Study
Source: PLoS One. 2007 Oct 10;2(10):e975. doi: 10.1371/journal.pone.0000975 (PMC1995762; doi:10.1371/journal.pone.0000975)
Supplement: Table S1 — (0.06 MB DOC) [file pone.0000975.s001.doc]

**TABLE S1.** Association of EPC number and colony number with common carotid artery IMT and carotid artery arteriosclerosis score.

| Ultrasound measure* | tertile group for EPC number | | |  |  |  |
| --- | --- | --- | --- | --- | --- | --- |
| **EPC number** | **1 (low)** | **2 (medium)** | **3 (high)** | **P for trend^†^** | **P for trend^‡^** | P for trend^§^ |
| Common carotid artery IMT (μm) | 1025 | 1008 | 1003 | 0.241 | 0.059 | 0.020 |
| Carotid artery atherosclerosis score (mm) | 5.4 | 5.1 | 5.0 | 0.828 | 0.297 | 0.059 |
|  | tertile group for EPC colony number | | |  |  |  |
| EPC-CFUs number | **1 (low)** | **2 (medium)** | **3 (high)** | **P for trend^†^** | **P for trend^‡^** | P for trend^§^ |
| Common carotid artery IMT (μm) | 1009 | 1019 | 1004 | 0.610 | 0.625 | 0.739 |
| Carotid artery atherosclerosis score (mm) | 4.8 | 5.4 | 4.9 | 0.967 | 0.754 | 0.627 |

* Values presented are sex- and age-adjusted means.

† The model included variables for age (years) and sex (female, male).

‡ The multivariate model included the variables age (years), sex (female, male), smoking (number of cigarettes smoked daily), alcohol consumption (g/day), diabetes (no, yes), body mass index (kg/m^2^), HDL and LDL cholesterol (mg/dL), ln-transformed triglyceride level (mg/dL), ln-transformed hs-CRP level (mg/L), ln-transformed Lp(a) level (mg/dL), ln-transformed urinary ACR (ratio), ferritin concentration (μg/L), and systolic blood pressure (mmHg).

§ This model was additionally adjusted for all types of medication listed in table 1.
