# Supplementary material for: Endothelial Progenitor Cells, Cardiovascular Risk Factors, Cytokine Levels and Atherosclerosis – Results from a Large Population-Based Study
Source: PLoS One. 2007 Oct 10;2(10):e975. doi: 10.1371/journal.pone.0000975 (PMC1995762; doi:10.1371/journal.pone.0000975)
Supplement: Text S1 — (0.07 MB DOC) [file pone.0000975.s002.doc]

***Online Supporting Text***

# Title page

# Endothelial progenitor cells, cardiovascular risk factors, cytokine levels and atherosclerosis – results from a large population-based study

Qingzhong Xiao^1^*, Stefan Kiechl^2^*, Seema Patel^1^, Friedrich Oberhollenzer^3^*,* Siegfried Weger^2^, Agnes Mayr^4^, Bernhard Metzler^5^, Markus Reindl^2^, Yanhua Hu^1^, Johann Willeit^2^ and Qingbo Xu^1^

^1^Cardiovascular Division, King’s College London, University of London, London, UK; ^2^Department of Neurology (S.K.; S. W., M.R., J.W.) and ^5^Internal Medicine (B.M), Medical University Innsbruck, Innsbruck, Austria; ^3^Departments of Internal Medicine (F.O.) and ^4^Laboratory Medicine (A.M.), Hospital of Bruneck, Italy;

*Authors contributed equally

Running Title: EPC in a general population

Word Count: 6200

Correspondence to:

Professor Qingbo Xu,

Cardiovascular Division, King’s College London, University of London,

Bessemer Road, Denmark Hill Campus, London, SE5 9PJ, UK

Tel: +44 20 3299 1488

Fax: +44 20 3299 3510

Email: [qingbo.xu@kcl.ac.uk](mailto:qingbo.xu@kcl.ac.uk)

**Methods**

**Immunofluorescent staining**. The procedure used for immunofluorescent staining was similar to that described previously(1). Antibodies against CD34 (CD34-FITC, Miltenyi Biotec, Germany), VE-cadherin (Santa Cruz Biotech., Santa Cruz, CA, USA), AC133 (Miltenyi Biotec, Germany), KDR (KDR-PE, R&D System, UK), and vWF (Santa Cruz Biotech.) were used. Cell nuclei were then counter stained with DAPI and examined with a fluorescence microscope (ZEISS, Axioplan 2 imaging).

**Results**

**Characterization of EPC-CFU and EPC:** Colonies were only counted as EPC-CFU if they consisted of more than 50 cells and contained a core of rounded cells with flat, spindle shaped cells emanating from the periphery (**Figure S1A**). Floating cell clumps or adherent cell clumps without any extending flat, spindle cells were ignored (**Figure S1B**). The average of colony numbers from more than three separate wells was documented. Surprisingly, the effects of different source and batch of serum used and if the complete medium applied is fresh or old on EPC colony formation are hugely. We observed that much less of EPC-colonies were grown in some serum, however, much more EPC-colonies were emerged in other serum in our preliminary experiment. We also found that much more EPC-CFUs were observed in fresh complete medium than that of old one. Such issues to choice optimal method to analyze the EPC-CFU and keep all the assay conditions consistent in whole study should be strongly addressed in future EPC-CFU experiment. Data shown in **Figure S1C** indicated that cells composing EPC-CFU uptook DiI-Ac-LDL. To further confirm that these colonies were real EPC colonies, cells within colony were taken out and dissociated with trypsin-EDTA buffer, then re-plated onto chamber slide and cultured overnight, followed by double immunofluorescent staining with antibodies against KDR, AC133, and CD34. Almost all the cells derived from EPC-CFU were double positive for AC133 and KDR (**Figure S1D to S1F**), or CD34 and KDR (**data not shown**), which indicated that these colonies were real EPC colonies. In this study, double positive cells for DiI-Ac-LDL and Lectin were counted as EPC (**Figure S1G to S1I,** arrow), and recorded in EPC culture assay. Quantitative analysis of the ratio of EPC to all adherent cells (blue nuclear) per high field implied that approximately 75±15% of cells were EPC in this stage. In the preliminary study, some slides were subjected to immunofluorescent staining with antibodies against KDR, AC133, CD34, VE-cadherin, and vWF to investigate these markers expression on EPC. We found that most of them were double positive for KDR and AC133 (**Figure S1J to S1L**), as well as positive for CD34, VE-cadherin, and vWF (**data not shown**). Further quantitative analysis indicated that the percentage of KDR and AC133 double positive cells was 70.5±13.7%, which was similar to that of DiI-Ac-LDL and Lectin double positive cells. According to these data and previous publications(2-6), this method was used in the following experiments to analyze the EPC numbers in circulation.

**Figure Legends**

**Figure S1.** Characterisation of EPC-CFU and EPC. Representative images of EPC-CFU **(A)**, cell clumps (non-EPC-CFU) **(B)**, and DiI-Ac-LDL taken up by cells within EPC-CFU **(C)** was presented here. **(D to F)** Representative images show that cells derived from EPC-CFU are double positive for KDR and AC133. Representative images **(G to I)** show that EPC are double positive for DiI-Ac-LDL and Lectin. Arrow indicates EPC. Representative images **(J to L)** show that EPC are double positive for KDR and AC133. Nuclear were counterstained with DAPI (blue).

**Figure S2.** **Panel A** is a distribution chart of EPC and EPC-CFU numbers (per 10^6^ PBMNC). **Panel B** shows the decline of EPC and EPC-CFU numbers (per 10^6^ PBMC) with age.

**References:**

**1. Xiao, Q., Zeng, L., Zhang, Z., Margariti, A., Ali, Z. A., et al. (2006) Sca-1+ progenitors derived from embryonic stem cells differentiate into endothelial cells capable of vascular repair after arterial injury. *Arterioscler Thromb Vasc Biol* 26, 2244-2251**

**2. Urbich, C., Heeschen, C., Aicher, A., Dernbach, E., Zeiher, A. M., et al. (2003) Relevance of monocytic features for neovascularization capacity of circulating endothelial progenitor cells. *Circulation* 108, 2511-2516**

**3. Vasa, M., Fichtlscherer, S., Adler, K., Aicher, A., Martin, H., et al. (2001) Increase in Circulating Endothelial Progenitor Cells by Statin Therapy in Patients With Stable Coronary Artery Disease. *Circulation* 103, 2885-2890**

**4. Aicher, A., Heeschen, C., Mildner-Rihm, C., Urbich, C., Ihling, C., et al. (2003) Essential role of endothelial nitric oxide synthase for mobilization of stem and progenitor cells. *Nat Med* 9, 1370-1376**

**5. Seeger, F. H., Haendeler, J., Walter, D. H., Rochwalsky, U., Reinhold, J., et al. (2005) p38 mitogen-activated protein kinase downregulates endothelial progenitor cells. *Circulation* 111, 1184-1191**

**6. Chavakis, E., Aicher, A., Heeschen, C., Sasaki, K., Kaiser, R., et al. (2005) Role of beta2-integrins for homing and neovascularization capacity of endothelial progenitor cells. *J Exp Med* 201, 63-72**
