# Supplementary figures and images for: Endothelial Progenitor Cells, Cardiovascular Risk Factors, Cytokine Levels and Atherosclerosis – Results from a Large Population-Based Study
Source: PLoS One. 2007 Oct 10;2(10):e975. doi: 10.1371/journal.pone.0000975 (PMC1995762; doi:10.1371/journal.pone.0000975)

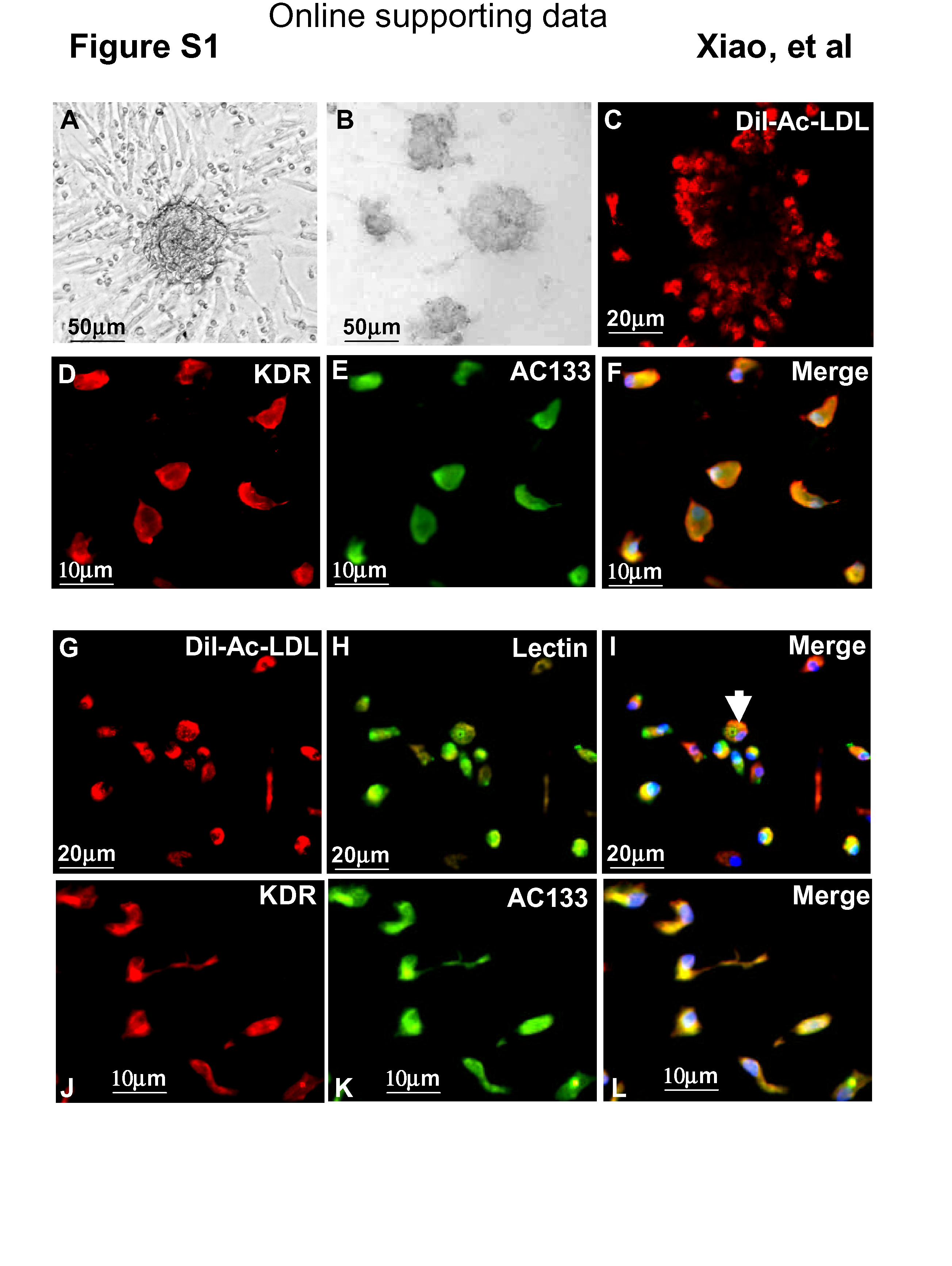

Supplement: Figure S1 — Characterisation of EPC-CFU and EPC (2.88 MB TIF) [file pone.0000975.s003.tif]

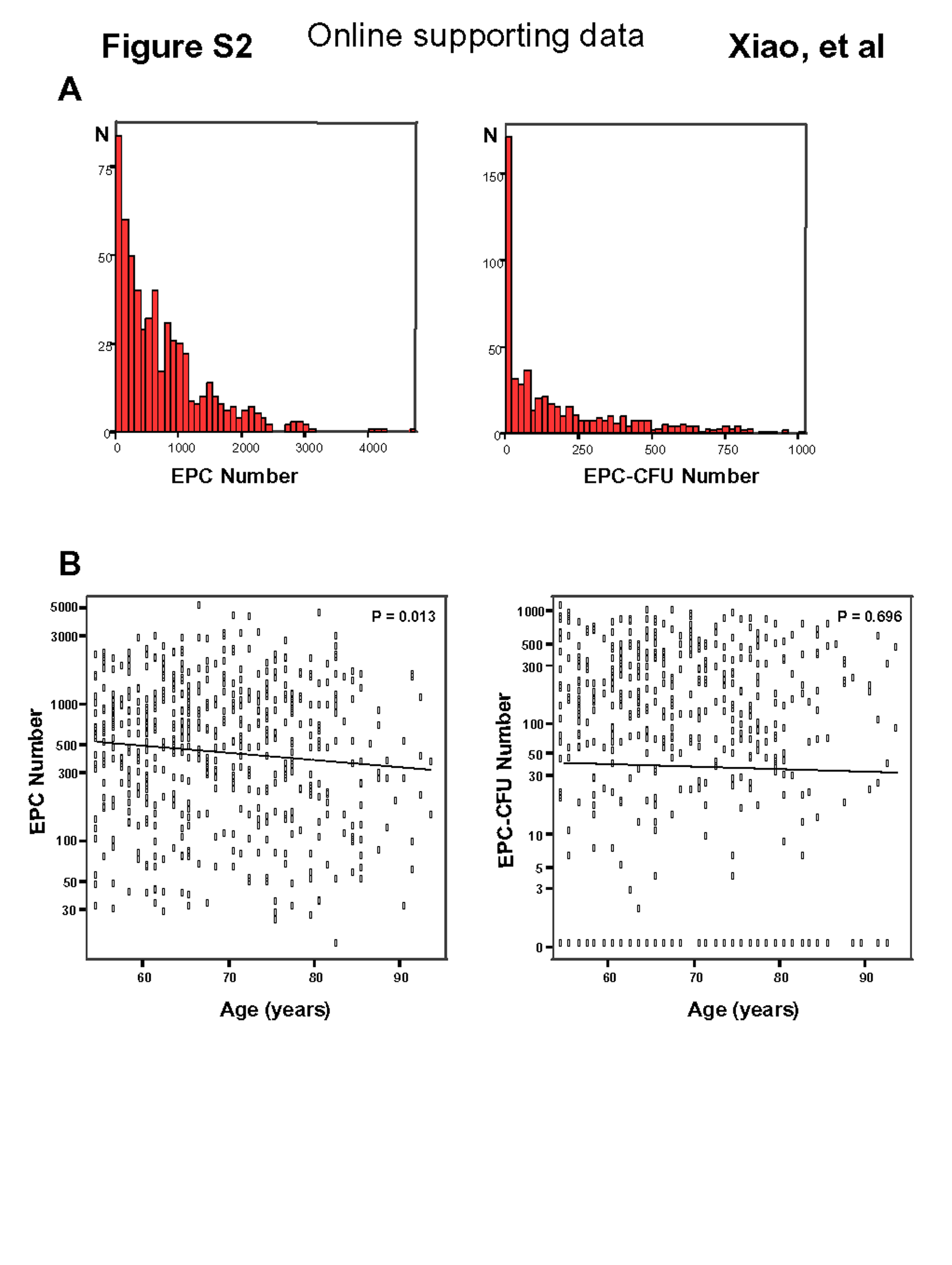

Supplement: Figure S2 — Distribution of EPC and EPC-CFU, and the decline of EPC and EPC-CFU numbers with age. (1.17 MB TIF) [file pone.0000975.s004.tif]
